# Gold Nanoparticles-Enhanced Gene Transfer Driven by MHz-Frequency Nanosecond Pulsed Electric Fields

**DOI:** 10.3390/biom15121736

**Published:** 2025-12-13

**Authors:** Veronika Malyško-Ptašinskė, Eivina Radzevičiūtė-Valčiukė, Anna Szewczyk, Barbora Lekešytė, Paulina Malakauskaitė, Eglė Mickevičiūtė-Zinkuvienė, Augustinas Želvys, Natalija German, Julita Kulbacka, Vitalij Novickij

**Affiliations:** 1 Faculty of Electronics, Vilnius Gediminas Technical University, LT-10223 Vilnius, Lithuania; eivina.radzeviciute@imcentras.lt (E.R.-V.);; 2 Department of Immunology and Bioelectrochemistry, State Research Institute Centre of Innovative Medicine, LT-08406 Vilnius, Lithuania; 3 Department of Molecular and Cellular Biology, Faculty of Pharmacy, Wroclaw Medical University, 50-367 Wroclaw, Poland

**Keywords:** electroporation, nanosecond, high-frequency, gold nanoparticles, GFP, gene electrotransfer, in vitro

## Abstract

Electroporation can be used as an effective non-viral gene delivery method, while the application of conductive nanoparticles (NPs) with pulsed electric fields (PEFs) may increase treatment efficacy due to local field amplification in close proximity to the cell plasma membrane. In this work, we have employed 100 ns and 300 ns pulses (9–17 kV/cm), which were delivered in bursts (n = 100) and predefined inter-pulse delays (100–900 ns), which enabled successful gene delivery (4.7 kbp; p-EGFP-N1) using pulses as short as 100 ns, which previously was considered impossible. As a model, a murine breast cancer cell line (4T1) was used. It was shown that sub-microsecond pulses (i.e., 300 ns) can be effective for gene delivery, whereas 100 ns pulses are several-fold inferior, yet still trigger successful gene transfer (>10% of cells being electrotransfected). In order to increase the efficacy of the treatment, we used gold nanoparticles (AuNPs; the diameter of 13 nm), which allowed us to achieve electrotransfection efficacy several-fold for both sub-microsecond and microsecond protocols (1.2 kV/cm × 100 µs × 8 pulses at 1 Hz). The results suggest high potential applicability of conductive nanoparticles in future translational or clinical research involving electroporation and gene transfer.

## 1. Introduction

Electroporation (EP) is a well-established method in various fields of application, from the food industry [1,2] to medical applications for permeabilization of the cell plasma membrane using pulsed electric fields (PEFs). These PEFs induce a transmembrane potential (TMP) across the lipid bilayer, resulting in the formation of aqueous nanopores in the cell membrane structure [3,4]. Typically for mammalian cells, EP is triggered when the induced TMP exceeds a threshold [5], corresponding to an external electric field strength of ~1000 V/cm for 100 μs pulses [6,7,8]. Depending on pulse intensity and duration, this can result in reversible electroporation (RE)—where pores reseal and cells survive—or irreversible electroporation (IRE), where excessive pore formation disrupts homeostasis and leads to cell death [9,10,11,12]. RE is particularly valuable for gene electrotransfer (GET), as it allows nucleic acids to enter viable cells. The technique has been widely applied across various cell types, including mammalian cells [13,14] due to its operational simplicity, efficiency, and broad applicability.

GET has broad applications in fundamental research, biotechnology [1,15], and clinical settings [16,17]. Its main uses include gene therapy for treating genetic disorders [18,19], cancer immunotherapy [20] through DNA vaccines [21], and delivery of therapeutic nucleic acids [22,23] for enhancement of the effects of tumor antigens [24]. For protocol optimization, green fluorescent protein (GFP)-encoding plasmids are commonly used to evaluate GET efficiency [19,25], whereas IL-12 [26] or GM-CSF [27] plasmids are used in more applied research, such as gene vaccination. The success of GET relies on various biological factors, including membrane composition [28], electroporation buffer [29], cell size and type [30,31], physiological conditions [32,33,34], as well as PEF properties. Standard GET protocols use square-wave pulses from tens of microseconds to milliseconds, at 100–1500 V/cm with 1–10 pulses [35,36,37]. Longer pulses are associated with higher membrane damage [38], increased muscle contractions (in the clinical setting) [39], and generate higher levels of reactive oxygen species (ROS).

Shorter pulses, e.g., nanosecond pulses (nsPEFs), can also be used for GET. The shorter pulse duration can reduce muscle excitation and minimize patient discomfort (i.e., one contraction instead of multiple) [40]. Additionally, plasmids are very sensitive to oxidative damage, whereas shorter pulses ensure lower ROS generation when compared to micro-millisecond procedures [41]. Furthermore, the higher frequency component of the burst contributes to tissue bioimpedance mitigation, potentially resulting in a more uniform treatment [42]. A key challenge lies in the lack of sufficient electrophoretic transport, which is critical for driving negatively charged DNA into permeabilized cells [43]. Consequently, nsPEF-based GET often results in low transfection efficacy unless supplemented with longer microsecond to millisecond range pulses (low voltage) [44]. Sub-100 ns pulses delivered at low frequencies (1–10 Hz) also fail to significantly enhance GET [45]. To fully exploit the benefits of nsPEFs-based GET, these limitations must be addressed through careful optimization of protocols.

Recent advancements have explored the use of high-frequency nsPEFs. Studies show that nanosecond pulses delivered at MHz repetition rates can achieve comparable or superior transfection efficiencies when compared to 100 μs pulses [46,47]. Such protocols trigger the phenomenon of residual transmembrane potential (TMP) accumulation—i.e., the cells have insufficient time to depolarize between consequent pulses in a burst—and thus, the size and stability of the pores are improved [48] during the burst. Additionally, the residual TMP throughout the burst can be modulated by pulse repetition rate; therefore, it is possible to maintain it in the reversible electroporation range without triggering IRE.

Gold nanoparticles (AuNPs) have attracted significant interest due to their high electrical conductivity [49], biocompatibility, and low toxicity [50]. Huang et al. have demonstrated that a combination of AuNPs with electroporation can significantly enhance the transfection of DNA and small interfering RNA efficiency [51]. The AuNPs amplify the PEFs in close proximity to the cell membranes, ensuring better permeabilization at lower fields, which is beneficial for nanosecond PEF protocols since typically they require amplitudes in the range of tens of kV/cm, which introduces engineering and safety problems in vivo or in clinics. Previously, we have demonstrated both in vitro and in vivo that bleomycin-based electrochemotherapy can be significantly potentiated by this approach [52,53,54].

The present study investigates the effects of 100–300 ns PEFs and varying interpulse delays (100–900 ns) on cell membrane permeabilization and GET efficiency, with and without AuNP-assisted field amplification. The aim is to show proof of concept that AuNPs can be successfully used for GET efficiency improvement.

## 2. Materials and Methods

**Figure 1:**
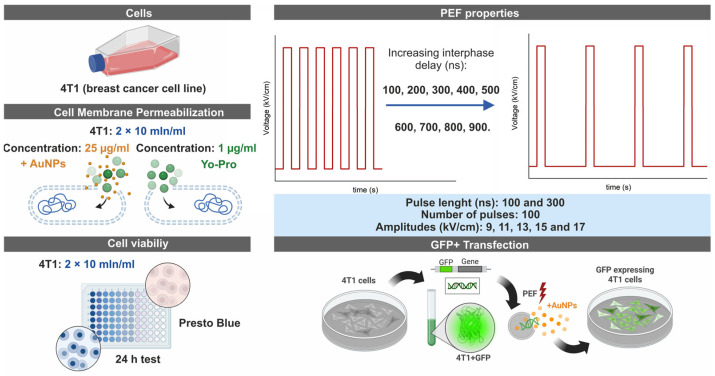
Simplified experimental scheme of methods involved in the paper.

The study focused on in vitro research of extremely high repetition frequency (>800 kHz) electroporation protocols (9–17 kV/cm × 100/300 ns × 100 pulses) for electrotransfection of cancer cells (4T1 cell line) and evaluation of the possibility to use gold nanoparticles (the diameter of 13 nm) for potentiation of the nanosecond pulses efficacy. The research covered cell plasma membrane permeabilization experiments (YO-PRO-1), analysis of the effects of inter-pulse delay (100–900 ns, without increasing input energy) on the outcome of the experiment, and viability assessment followed by in vitro gene delivery (4.7 kbp; p-EGFP-N1). The simplified experimental scheme is presented in Figure 1.

The detailed methodological procedures are overviewed further in the paper.

### 2.1. Cells

The murine 4T1 cell line (ATCC-CRL-2539, Manassas, VA, USA), originating from a spontaneous mammary tumor in BALB/c mice, was propagated in RPMI-1640 culture medium enriched with L-glutamine, 100 U/mL penicillin, 100 µg/mL streptomycin, and 10% fetal bovine serum (FBS). Cell cultures were sustained at 37 °C in a humidified atmosphere containing 5% CO_2_ to ensure optimal growth conditions. All media components and supplements were sourced from Gibco, Thermo Fisher Scientific (Waltham, MA, USA).

Before initiating experimental protocols, adherent cells were enzymatically released from culture surfaces using Trypsin-EDTA solution (Thermo Fisher Scientific, Waltham, MA, USA) and incubated for approximately 3–10 min until full detachment was achieved. The detached cells were subsequently collected by centrifugation and resuspended in electroporation buffer to prepare for further manipulation. For gene electrotransfer procedures, cell suspensions were standardized to a final density of 6 × 10^6^ cells per milliliter. In contrast, experiments assessing plasma membrane permeabilization and post-electroporation cell survival were conducted using suspensions adjusted to 2 × 10^6^ cells per milliliter.

### 2.2. Electroporation Setup and Parameters

Electric pulses were applied using a high-voltage pulse generator capable of producing waveforms with durations between 65 nanoseconds and 100 microseconds and output amplitudes of up to 3 kV. The generator also supported the delivery of pulse bursts at predefined frequencies ranging from 1 Hz to 5 MHz [55]. Electric pulses were delivered using a commercially available electroporation cuvette with a 1 mm gap size (Bio-Rad, Hercules, CA, USA), which served as a load of the generator.

To examine the effects of nanosecond electroporation employing 100 and 300 ns pulses compressed to a MHz burst, we varied the inter-pulse interval of unipolar pulses from 100 to 900 ns in increments of 100 ns. Correspondingly, the pulse repetition frequency for 100 ns pulses ranged from 5 to 1 MHz, and 2.5 to 0.83 MHz for 300 ns pulses. To identify the optimal electric field strength ensuring effective membrane permeabilization, pulse amplitudes in the range of 9–17 kV/cm were involved in the study (Figure 1). All nanosecond pulse protocols consisted exclusively of 100 unipolar pulses. As a baseline for reversible EP efficiency, the standard ESOPE protocol (1.2 kV/cm × 100 µs × 8 pulses at 1 Hz) was included in each experimental assay to serve as a reference.

### 2.3. Cell Permeabilization Detection Assay Using Yo-Pro-1

Membrane permeabilization following electroporation was evaluated by flow cytometry using a BD Accuri C6 cytometer (BD Biosciences, San Jose, CA, USA) and the fluorescent dye Yo-Pro-1 (YP1; Thermo Fisher Scientific, Waltham, MA, USA). Prior to pulsing, cells suspended in electroporation buffer were incubated with YP1 to reach a final dye concentration of 1 μM. For samples containing gold nanoparticles, 13 nm AuNPs were added to the electroporation buffer at a final concentration of 25 μg/mL. Subsequently, 50 μL of the stained suspension was transferred into an electroporation cuvette and exposed to the designated pulsing conditions. Following electrical treatment, all samples were kept at room temperature for 10 min to allow dye uptake, after which 150 μL of 0.9% sodium chloride solution was added to dilute the suspension. Fluorescence intensity of YP1 (excitation/emission maxima: 491/509 nm) was then measured in the FL1 detection channel equipped with a 533/30 nm bandpass filter. Data acquisition and post-processing were carried out using FlowJo analysis software (Version 10.8.2, BD, Becton Drive, Franklin Lakes, NJ, USA). The gating strategy was performed according to previous studies [56].

### 2.4. Viability Assay

Cell viability following treatment with pulsed electric fields was determined using the PrestoBlue cell metabolic activity assay (Thermo Fisher Scientific, Waltham, MA, USA). Measurements were performed 24 h post-treatment to evaluate the cytotoxic effects of each condition. A prepared suspension of 4T1 cells, with or without AuNPs, was placed between the electrodes for treatment under various PEF conditions. Following treatment, cells were seeded into 96-well flat-bottom microplates (TPP, Trasadingen, Switzerland).

After an initial incubation period of 10 min, 150 μL of complete growth medium was added to each well, and the plates were further incubated for 24 h under standard culture conditions. After 24 h of cultivation, the medium was removed, and the wells were rinsed twice with phosphate-buffered saline (PBS). Subsequently, 90 μL of PBS and 10 μL of the PrestoBlue cell viability reagent were dispensed into each well, followed by a 2 h incubation at 37 °C. Fluorescence intensity (excitation 540/20 nm; emission 620/40 nm) was then recorded using a Synergy 2 microplate reader operated with Gen5 software (Version: v3.0x, PN 5321002, BioTek, Shoreline, WA, USA).

### 2.5. Gene Delivery Experiments

The efficiency of gene delivery via electroporation was examined using the pEGFP-N1 plasmid (4.7 kbp), which encodes green fluorescent protein (GFP). For each experiment, 30 μL of cell suspension was mixed with 4 μL of plasmid DNA prepared with the HiPure Expi Plasmid Gigaprep Kit (Thermo Fisher Scientific, Waltham, MA, USA), and all handling was performed on ice. Pulsed electric field (PEF) treatments were applied under multiple protocols, both in the presence and absence of AuNPs.

Following a brief 10 min period for initial recovery, 500 μL of RPMI medium was added to each well, and the plate was incubated at 37 °C in a 5% CO_2_ humidified atmosphere for 24 h. After incubation, cells were detached using trypsin, collected into microcentrifuge tubes, centrifuged, and resuspended in 90 μL of PBS. The cell suspensions were then loaded into 96-well round-bottom plates for flow cytometric analysis using both the BD Accuri C6 and the Amnis FlowSight cytometers (Luminex, Northbrook, IL, USA). GFP expression was detected using the FL1 channel (533/30 nm bandpass filter) on the Accuri C6 and channel 2 (532/55 nm) on the FlowSight. For a detailed description of the electrotransfer procedure, please refer to our previous work [52,56].

### 2.6. The Preparation of AuNPs

A red-colored colloidal solution of AuNPs (50 μg/mL) with an average diameter of 13 nm was synthesized in accordance with the established protocol reported in the literature [57]. In brief, placed in two different flasks— 0.0125% tetrachloroauric (III) acid (Sigma-Aldrich, Co., St. Louis, MO, USA) solution and a solution comprising 0.2% trisodium citrate dihydrate (Sigma-Aldrich, Oakville, ON, Canada), 0.00125% tannic acid (Carl Roth GmbH + Co., Karlsruhe, Germany)—were heated to 60 °C with continuous magnetic stirring. All reagents used for the synthesis of AuNPs were of high purity. Subsequently, both solutions were mixed, heated until they boiled under continuous stirring, and left to boil for a few minutes. Thereafter, the flask with the solution of AuNPs was transferred into the ice to complete the reduction process and subsequently stored in a refrigerator prior to further measurements. The synthesized AuNPs colloidal solution (pH 6.5) was characterized by 0.9481 a.u. of an optical density at 520 nm of a wavelength and −34 mV of Zeta potential.

### 2.7. Statistical Analysis

Data were analyzed using a one-way analysis of variance (ANOVA), with statistical significance set at *p* < 0.05. Following detection of significant effects by ANOVA, pair-wise comparisons between groups were conducted using Tukey’s Honestly Significant Difference (HSD) test to identify specific differences, applying the same significance criterion (*p* < 0.05). All statistical analyses were performed using OriginPro software (version 18.0; OriginLab, Northampton, MA, USA). Results are presented as mean ± standard deviation (SD), based on at least three independent replicates.

## 3. Results

### 3.1. Cell Membrane Permeabilization

**Figure 2:**
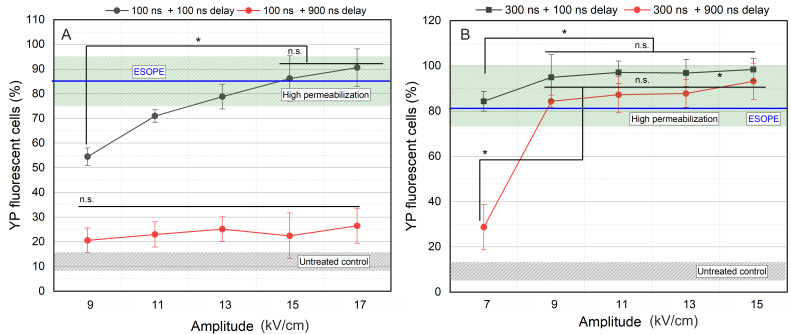
The dependence of cell membrane permeabilization on applied pulsed electric field, where (**A**) 100 ns pulses and (**B**) 300 ns pulses. ESOPE refers to 1.2 kV/cm × 100 µs × 8, 1 Hz protocol. Data presented as mean ± SD (n ≥ 3). One-way ANOVA: Asterisk (*) *p* < 0.05, n.s., *p* > 0.05. Mean ± SD of the untreated control samples represented in the gray hatched area. The green hatched area refers to a high permeabilization rate, i.e., >75%.

Firstly, cell membrane permeabilization was assessed at marginal repetition rates of nanosecond pulses involved in the study (100 and 900 ns). The 100 and 300 ns pulses were delivered in a burst of 100 pulses, and the efficacy of YP delivery is summarized in Figure 2.

As can be seen in Figure 2A, interpulse delay has a dramatic influence on the cell membrane permeabilization when pulses as short as 100 ns are used. Independent of the amplitude (9–17 kV/cm), 100 ns pulses do not trigger high permeabilization if the delay between the pulses is 900 ns. However, in the case of 5 MHz pulses (100 ns delay), an expected dose-dependent curve was acquired. High permeabilization is triggered when 13 kV/cm PEFs were used, while 15 and 17 kV/cm induced ESOPE-equivalent permeabilization.

For 300 ns pulses (Figure 2B), all 7–15 kV/cm protocols induced high permeabilization when the 100 ns inter-pulsed delay was used. The 7 kV/cm protocol was not effective if the delay was increased to 900 ns.

Considering the results, further study was limited to two amplitudes from each pulse duration to characterize the effects of repetition frequency on the permeabilization efficacy. Therefore, the 15 and 17 kV/cm PEFs for 100 ns pulses and 9 and 11 kV/cm PEFs for 300 ns pulses were selected.

**Figure 3:**
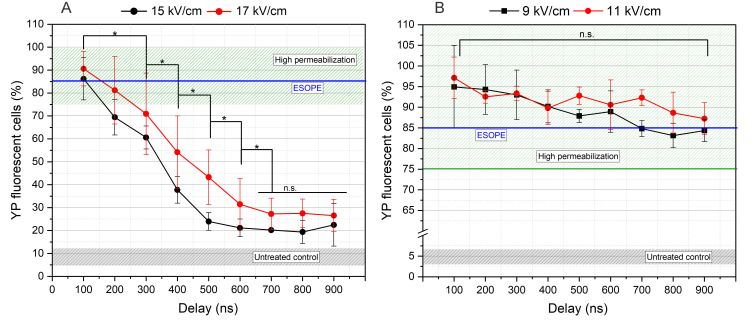
The dependence of cell membrane permeabilization on PEFs inter-pulse delays, where (**A**) 100 ns pulses and (**B**) 300 ns pulses. ESOPE refers to 1.2 kV/cm × 100 µs × 8, 1 Hz protocol. Data presented as average ± SD (n ≥ 3). One-way ANOVA: Asterisk (*) *p* < 0.05, n.s., *p* > 0.05. The average value of YP fluorescent cells within untreated control samples received no PEFs represented in gray hatching. Green hatching refers to a high permeabilization range above 75%.

Subsequently, we have varied the inter-pulse delay with steps as low as 100 ns and characterized the effects on cell membrane permeabilization. The results for each pulse duration are summarized in Figure 3. The goal was to modulate the burst frequency without altering other parameters to understand the effects of pulse repetition frequency on electroporation.

A clear dependency on inter-pulse delay was observed with protocols employing 100 ns pulses. It was evident that once inter-phase delay exceeded 100 ns (corresponding to frequencies below 5 MHz)—a marked reduction in the percentage of YP-positive cells at both tested field strengths was observed. The differences between 15 and 17 kV/cm are not statistically significant in many cases; however, on average, the higher amplitude triggers a higher permeabilization, which is an expected result. For GET experiments, the 15 kV/cm × 100 ns × 100 delivered at 5 MHz repetition frequency was selected since it induced the same permeabilization efficacy as the 17 kV/cm protocol. It is concluded that application of 100 ns pulses with lower pulse repetition frequencies (e.g., <3 MHz) has no practical value unless the PEFs amplitude is further increased, which may be a challenge in clinical or in vivo settings.

In case of 300 ns pulses, the dependence on inter-pulse delay is less profound; however, the tendency of reduction in the number of permeabilized cells can also be seen if the delay between pulses is increased. The GET experiments were limited to the 9 kV/cm PEFs for this pulse duration; however, both 100 and 900 ns inter-pulse delay protocols were chosen since both induce ESOPE-equivalent permeabilization efficacy. The 11 kV/cm was not used, since it involves more than 60% higher energy input when compared to the 100 ns protocols. However, the energy input of 15 kV/cm × 100 ns and 9 kV/cm × 300 ns pulses is comparable (<10% difference).

### 3.2. Cell Viability with and Without AuNPs

**Figure 4:**
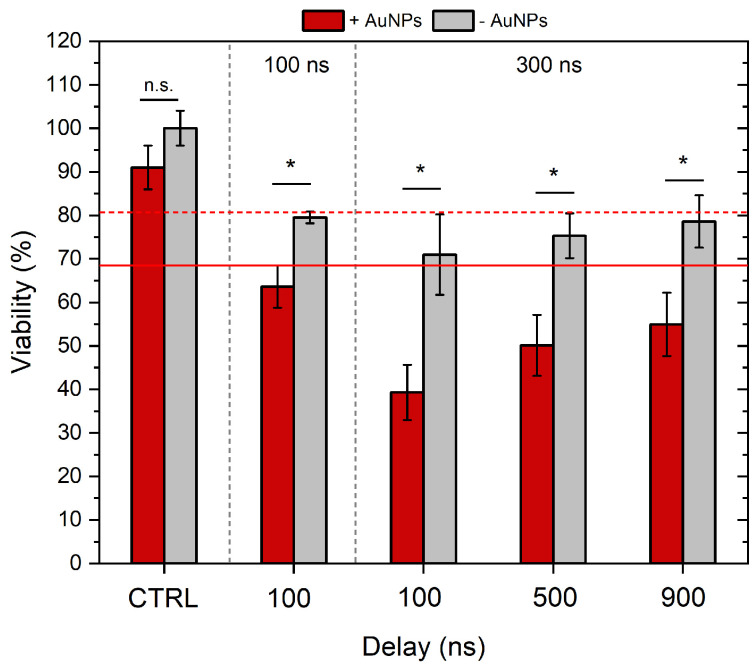
The dependence of cell viability on applied electric field parameters and gold nanoparticles. Red dashed and non-dashed lines show ESOPE treatment without and with AuNPs, respectively. ESOPE refers to 1.2 kV/cm × 100 µs × 8, 1 Hz protocol. One-way ANOVA: Asterisk (*) *p* < 0.05, n.s., *p* > 0.05. Data represents the average ± SD of at least three replicates per protocol (n ≥ 3).

Successful GET requires a high degree of permeabilization, typically indicated by YP uptake > 75% of the cell population. At the same time, maintaining high viability post-treatment is essential. Therefore, in this step, we characterized cell viability with the selected 100 and 300 ns protocols and combined the treatment with AuNPs. The ESOPE protocol (1.2 kV/cm × 100 μs × 8) served as reference. The results are summarized in Figure 4.

As can be seen, all the nanosecond protocols induced an ESOPE-equivalent drop in viability (~20%). The 300 ns pulses, when delivered with 100 ns inter-pulse delay on average, resulted in lower viability; however, the difference was not statistically significant. The application of AuNPs significantly reduced the viability of cells post-treatment, which is an expected result. In the case of the highest intensity protocol (300 ns protocol, with 100 ns delay), the viability dropped by more than 60%, which makes the protocols hardly applicable for GET applications. Therefore, further GET experiments were limited to the inter-pulse delay of 500 and 900 ns, which returned a viability of 50 and 55%, respectively.

As expected, the 100 ns protocol was the least cytotoxic out of nanosecond protocols, i.e., when combined with AuNPs, the viability of cells was ~63%. The 300 ns + 900 ns delay and the 100 ns + 100 ns delay protocols triggered ESOPE-equivalent viability without AuNPs (*p* > 0.05); however, all nsPEF protocols were more cytotoxic when combined with AuNPs. The 300 ns + 100 ns delay protocol was not further used in the study due to poor cell viability (~40%) when combined with AuNPs.

### 3.3. Gene Electrotransfer

**Figure 5:**
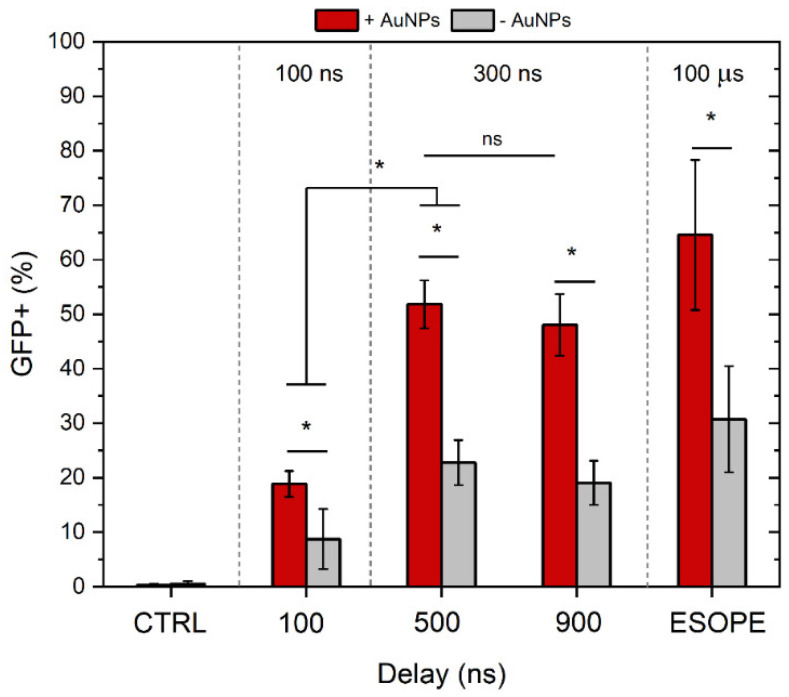
The dependence of GET efficiency on PEFs parameters and gold nanoparticles. ESOPE refers to 1.2 kV/cm × 100 µs × 8, 1 Hz protocol. CTRL—corresponds to untreated control. Data presented as mean ± SD (n ≥ 3). One-way ANOVA: Asterisk (*) *p* < 0.05, n.s., *p* > 0.05.

The gene electrotransfer experiments have been performed with the protocols described above separately and in combination with AuNPs. The ESOPE protocol (1.2 kV/cm × 100 μs × 8) served as a reference. The results are summarized in Figure 5.

As shown in Figure 5, the combination with gold nanoparticles significantly enhanced GET efficiency, as evidenced by more than a two-fold increase in the percentage of GFP-positive cells across all tested protocols, including ESOPE. Specifically, the application of 300 ns pulses with inter-pulse delays of 500 and 900 ns, in combination with AuNPs, resulted in approximately 50% GFP-positive cells. In contrast, corresponding protocols without AuNPs achieved only around 20% GFP expression. Notably, the ESOPE protocol with AuNPs on average resulted in the highest GET efficacy; however, the result was not statistically significant when compared to 300 ns protocols. At the same time, 100 ns protocols enabled the successful transfection of 4T1 cells; however, the efficiency rates were several-fold lower when compared to other protocols involved in the study.

As mentioned above, successful GET is a compromise between the fraction of cells being transfected and the overall cell viability. Therefore, to compare the protocols more objectively, we have summarized the efficiency of the treatment in Table 1.

As can be seen in Table 1, the highest percentage of cells being transfected out of the initial suspension (100%) is achieved using the ESOPE protocol. In all cases, AuNPs potentiate the GET treatment significantly (on average ~1.7-fold), which is an excellent result.

## 4. Discussion

The aim of the study was to determine the effects of inter-pulse delay on nanosecond electroporation efficiency over a range that had not been covered before. Additionally, we combined the most prominent protocols with AuNPs to potentiate the efficacy in the context of GET.

According to current knowledge, the application of ultra-short pulses (i.e., 100 ns or sub-100 ns) can hardly be used for GET due to the lack of an electrophoretic component, resulting in poor electransfer of DNA. The methodology can be improved with a combination of nsPEFs with longer pulses (e.g., millisecond ones) as proposed by Guo et al., similar to the HV/LV methodology [44]. However, in such a case, the benefits the nsPEFs offer are hardly exploited, and the limitations applicable previously would still be in place (e.g., muscle contractions, ROS, complex exposure systems, etc.). To overcome this problem, we tried to utilize burst compression and induction of the residual TMP accumulation phenomenon, which allows the successful application of nsPEFs pulses for molecular delivery. Nevertheless, the 100 ns pulses did not result in acceptable efficacy of GET—i.e., ~10% GET efficiency was induced, which still has limitations for practical applications, even though it is the first study to confirm that pulses as short as 100 ns can still enable GET. Based on the results, we hypothesize that the optimization of the 100 ns protocol is possible by increasing the amplitude further (>15 kV/cm). However, considering the context of the study and the limitations described above, it has limited practical value due to challenges in vivo to ensure such amplitudes. Compressing the pulses even further is also hardly possible, since semiconductor technology is already at its limit, and the pulses are delivered at a 50% duty cycle.

At the same time, the 300 ns pulses, when delivered at high frequency (500 and 900 ns delays), can induce ESOPE-equivalent efficacy of GET. Such a result was acquired at 9 kV/cm PEFs, which is applicable in vivo. Also, our results indicate that burst compression can further reduce the PEFs threshold for 300 ns pulses, which makes the strategy promising. It should also be noted that we were not able to acquire better GET with 300 ns pulses, when compared to our previous work [53]. Basically, ESOPE-equivalent efficacy can be derived both with 1 and 2.5 MHz frequencies. In order to further potentiate the treatment, other treatment parameters such as buffer composition or adjuvant treatment should be used. In this work, we attempted to use AuNPs, which showed very promising results both in vitro and in vivo in the context of ECT [54]. Based on the results of this work, it is also evident that AuNPs can be successfully used for GET. With all protocols involved (both microsecond and nanosecond), the GET efficacy increased significantly.

We used a single 4.7 kbp plasmid, and plasmid size strongly affects membrane translocation and intracellular transport; the effectiveness of the proposed protocols may vary with different plasmid sizes [58]. Conventional nsPEFs are generally insufficient for large molecules, as pulse durations are too short to ensure effective molecular delivery; however, high MHz repetition can accumulate residual TMP, extending the window for DNA entry. Also, experiments were conducted exclusively in 4T1 cells, which is a popular model cell line; however, other cell lines’ membrane composition and cytoskeletal organization can affect pore formation [59], stabilization, and resealing [60,61,62]. Investigating a broader range of plasmid sizes and different cell types will be important to determine the limits of this approach and evaluate its translational potential in future studies.

As a limitation, the drop in viability following the PEFs + AuNPs treatment could also be highlighted. While it is still evident that the drop in viability is effectively compensated by the increase in percentage of transfected cells (refer to Table 1), we still believe that optimization in terms of AuNPs structure, concentration, and PEFs parameters is possible. It is known that bigger nanoparticles or nanostructures, such as nanorods, have better potency for PEF-amplification [63]. Other strategies, including surface passivation (e.g., PEGylation [64] coating) to minimize nonspecific interactions, may be considered. Therefore, further research should focus on optimizing the nanotechnology underpinning the methodology.

Without optimization of AuNPs structure and characteristics, the efficacy of adjuvant treatments with PEFs + AuNPs is limited for gene transfer, according to Polajžer et al. [65]. The study concluded that nanoparticles do not substantially enhance membrane permeabilization, because the electric field amplification they induce is highly localized and several orders of magnitude smaller than the transmembrane field required for electroporation, which is not the case in our study. To improve AuNPs-assisted electroporation, several optimization pathways are possible. For instance, controlling particle size and geometry matters: larger or elongated particles produce stronger field enhancements, as predicted in FEM. Polajžer et al. show that spherical AuNPs of 10 nm radius produce smaller field peaks than larger ones, while elongated particles show more pronounced enhancement. The results of previous works and our study highlight the critical role of AuNPs themselves in inducing a synergistic effect with PEF rather than the PEF parameters. The effects attributed to nanoparticles may not be straightforward, as additional cellular processes can modulate the outcome. For example, AuNPs are known to interact directly with plasmid DNA [66]. They can also perturb intracellular ionic homeostasis by inducing calcium fluctuations that subsequently influence calcium-dependent potassium channels [67], indicating a mechanism that extends beyond direct membrane permeabilization. Such interactions may increase the likelihood of plasmid uptake once electroporation-induced pores form without necessitating any alteration in the permeabilization process itself. Thus, the enhanced GET efficiency observed in our study may also be partly influenced by AuNP–DNA interactions and downstream intracellular processing, rather than from nanoparticle-mediated changes to the biophysical parameters of electroporation. This hypothesis requires further justification in future studies.

The results of our work are also in agreement with insights by Ghorbel et al. that even if the local amplification of the electric field is low, conductive nanoparticles can play an important role in increasing the size or number of membrane pores during electroporation and thus the efficiency of the treatment [68]. Nevertheless, it is agreed that unless AuNPs are in very close contact with or partially embedded in the membrane, only modest amplification of the electric field is achieved. To ensure the position of conductive NPs near the cell membrane, either pulses involving a higher electrophoretic component are required (longer duration) or the treatment may rely more on initial surface chemistry and binding interactions. Thus, surface charge or functionalization is critical. Positively charged or membrane-targeting coatings (e.g., using peptides, transferrin, or cationic polymers [69,70,71]) increase binding to cell surfaces, reduce nanoparticle–membrane distances by adjusting zeta potential [72] or altering surface properties [73,74], and thereby potentially raise the local TMP.

Maintaining colloidal stability is equally important [75]. Aggregated or sedimented AuNPs can reduce molecular delivery uniformity due to changes in effective particle size, limit tissue penetration, or cause non-uniform electric field distribution. To avoid this, NPs stability in biological media must be preserved so that particle size, charge, and dispersion remain consistent during pulse exposure. Therefore, both modeling and experimental work should focus on optimizing NPs characteristics (such as size, shape, and functionalization for positive or targeting charge), ensuring colloidal stability in physiological conditions, and designing pulse protocols (pulse width, repetition rate, and burst) to enhance field effects and TMP control.

## 5. Conclusions

To summarize, nsPEFs can be successfully used for GET; however, the duration should be on the order of several hundred nanoseconds to ensure acceptable efficacy. It is possible to achieve GET even with ultrashort pulses such as 100 ns; however, the efficiency falls below standards for practical applications. It is also indicative that a further increase in PEFs amplitude is irrelevant in the context of 100 ns pulses due to technological challenges in vivo. At the same time, it can be concluded that modulation of the pulse repetition frequency is an excellent way to modulate nanosecond electroporation efficiency, which does not require an increase in energy input. Finally, it is concluded that AuNPs can enhance GET efficiency while remaining safe, biocompatible, and requiring no functionalization. Further optimization of nanoparticle size, form factor, or concentration is required to optimize the treatment.

## Figures and Tables

**Table 1:** The gene electrotransfer efficiency dependence on PEF protocols and gold nanoparticles.

PEF Protocol	Viability (%) × Transfection Efficiency (%)		
	Without AuNPs	With AuNPs	Improvement with AuNPs
15 kV/cm × 100 ns × 100 (delay 100 ns)	6.9%	12.0%	1.74-fold
9 kV/cm × 300 ns × 100 (delay 500 ns)	17.1%	26.0%	1.52-fold
9 kV/cm × 300 ns × 100 (delay 900 ns)	15.0%	26.4%	1.76-fold
1.2 kV/cm × 100 μs × 8 (delay 1 s)	24.9%	43.9%	1.76-fold

## Data Availability

The original contributions presented in this study are included in the article. Further inquiries can be directed to the corresponding authors.
